# 4.G. Workshop: Economic evaluations of public health interventions: from case studies to next methodological steps

**DOI:** 10.1093/eurpub/ckac129.234

**Published:** 2022-10-25

**Authors:** Organised by: EUPHA-ECO, EUPHA-HTA, Chair persons: João Vasco Santos (EUPHA-ECO), Vanessa Gorasso (EUPHA-ECO)

## Abstract

For establishing evidence-based or evidence-informed decision making in health systems, it is essential to determine which public health interventions and health policies are worth investing in, considering that public budgets are always limited. For that purpose, we can use well-established economic evaluation methods based on marginal analysis as cost-benefit and cost-effectiveness analyses. While the use of economic evaluations is a common practice in health technology assessment of drug and medical device in many high-income countries, the systematic assessment of public health interventions is marginal and non-compulsory. In fact, if conducted at all, many economic evaluations of public health interventions are performed internally without dissemination outside organisations. In general, there is already some guidance on how to perform and report economic evaluations that can also be used for public health interventions, as the recent update version of Consolidated Health Economic Evaluation Reporting Standards (CHEERS) check list. However, some methodological and implementation difficulties still persist, including insufficient or low-quality data on costs and outcomes (e.g. absence of experimental studies), complexities related to the need of measuring outcomes in the very long term an on aspects beyond the healthcare sector, or high relevance of contextual factors that are difficult to measure. This workshop will take on some examples of economic evaluations of public health interventions and explore how analyses, methods and implementation can be improved in order to allow a more evidence-based or evidence-informed decision making process.

**Key messages:**

• Economic evaluations of public health interventions could be performed more frequently when it comes to the decision making. Planning and implementation difficulties are highlighted in this workshop.

• Methods used in current economic evaluations of public health interventions can still be improved with a correct estimation of costs and outcomes, based on good quality data.

